# Toxicological evaluation of volatile organic compounds emitted from scented candles: *in silico* ADMET profiling, oxidative stress, inflammation, and lung injury in rats

**DOI:** 10.3389/fpubh.2025.1678549

**Published:** 2025-09-18

**Authors:** Mervat S. Mohamed, Awatif M. E. Omran, Amnah Obidan, Seham O. Alsulami, Nuha B. Aljohani, Eman H. Alshehri, Hajer Alfarteesh, Norah Bakheet Aljohani, Ayat G. Ali

**Affiliations:** ^1^Department of Chemistry, Biochemistry Speciality, Faculty of Science, Cairo University, Giza, Egypt; ^2^Department of Biochemistry, Faculty of Science, University of Tabuk, Tabuk, Saudi Arabia; ^3^Department of Biochemistry, College of Science, King Saud University, Riyadh, Saudi Arabia; ^4^Biology Department, King Khalid University, Abha, Saudi Arabia; ^5^King Abdul Aziz University Hospital, Jeddah, Saudi Arabia; ^6^Department of Biochemistry, El Sahel Teaching Hospital, GOTHI, Cairo, Egypt

**Keywords:** scented candles, ADMET, oxidative stress, inflammation, pulmonary toxicity

## Abstract

Scented candles are widely used in the Middle East, particularly in the Arab Gulf region, to enhance indoor environments. However, limited ventilation in enclosed air-conditioned spaces can cause emissions to accumulate, posing health risks. Although the chemical composition of candle emissions has been examined, their *in vivo* toxicological effects under realistic exposure conditions remain unclear. This study evaluated the toxicity of scented candle emissions in male Wistar rats. Fifty-four rats were divided into nine groups (*n* = 6/group): controls exposed to fresh air, unscented candle emissions, or scented candle emissions for 1, 3, or 6 h daily, 5 days per week for 8 weeks under indoor-like conditions. GC–MS analysis identified 20 volatile organic compounds (VOCs) in unscented and 60 in scented candles. In silico ADMET profiling predicted toxicity for several compounds. Biochemical assays showed elevated serum TNF-*α* and IL-6, increased MDA, and reduced CAT and T-SOD activities in lung tissue, indicating systemic inflammation and oxidative stress. qRT-PCR and immunohistochemistry confirmed upregulation of inflammatory markers (TNF-*α*, COX-2). Histopathology revealed inflammatory infiltration, fibrosis, and necrobiotic changes, particularly in scented candle-exposed groups. Chronic scented candle exposure in poorly ventilated spaces causes inflammation, oxidative stress, and lung injury.

## Graphical abstract

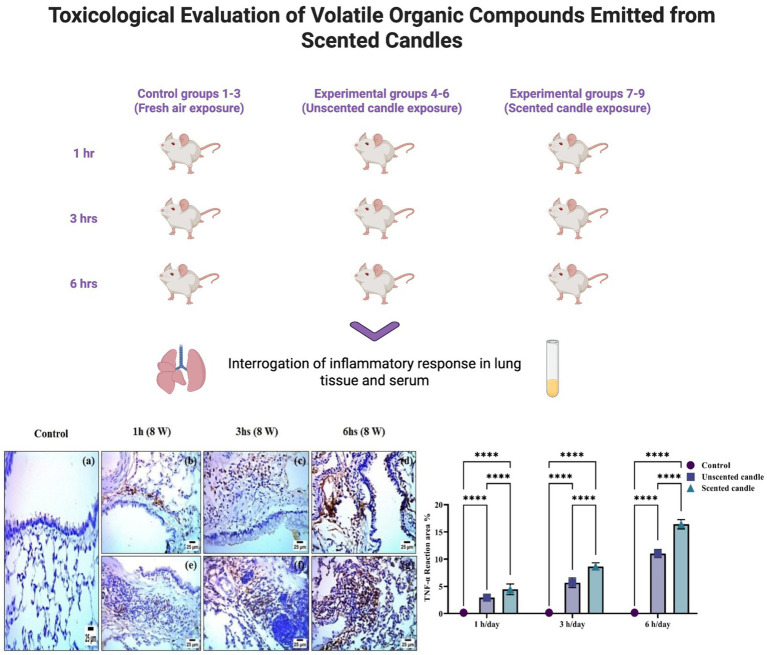

## 1 Introduction

Scented candles are widely used throughout the Middle East, particularly in the Arab Gulf region, for decorative purposes and to create pleasant indoor aromas in homes and offices. Odors play a fundamental role in human physiology and can significantly influence behavior and psychological states. Thus, the widespread use of scented candles represents not only a cultural tradition but also a practice with potential physiological and psychological implications (1). In the hot climate of this region, windows are often kept closed, and indoor environments rely heavily on-air conditioning, which typically lacks adequate fresh air exchange. This limited air exchange facilitates the accumulation of indoor air pollutants, particularly those emitted by scented candles. Under such enclosed conditions, burning scented candles can lead to a buildup of volatile organic compounds (VOCs), posing potential health risks. Since people spend a substantial amount of time indoors, understanding the impact of odor-emitting sources on indoor air quality is critically important (2).

Scented candles, like other common indoor sources such as furniture, construction materials, and fragrance-containing products, contribute to the overall burden of indoor emissions. These sources release complex mixtures of odorous chemicals, which can interact in unpredictable ways and affect human health (3). Such exposures have been associated with symptoms including eye, nose, and throat irritation, as well as headaches and general discomfort (4). Identifying the specific compounds responsible for these emissions and implementing mitigation strategies are essential steps toward improving indoor air quality (2). Accordingly, many studies have focused on characterizing key odorous compounds in indoor environments such as homes and offices (5).

Among these sources, scented candles are of particular concern due to their widespread use to mask unpleasant odors and create specific atmospheres, with the market for these products growing rapidly (6). Despite their popularity and accessibility, scented candles often lack regulatory oversight, and their raw materials are not always disclosed, which increasing the risk of harmful emissions during combustion (7). Moreover, no studies to date have used candles with known and standardized compositions specifically formulated for direct comparative analysis (8). Previous research has shown that burning scented candles releases combustion gasses—such as carbon monoxide and nitrogen oxides—that contribute to indoor air pollution (9). A wide range of aromatic substances may also be emitted, including aldehydes, hydrocarbons, alcohols, and polycyclic aromatic hydrocarbons (PAHs) like naphthalene, anthracene, and pyrene, which are known or suspected carcinogens (10). Exposure to scented candle fumes has been associated with a variety of health concerns (11). For example, animal studies simulating restaurant kitchen environments have demonstrated that combined exposure to candle fumes and psychological stress can exacerbate cardiopulmonary injury (12). Other research suggests a possible link between scented candle emissions and increased bladder cancer risk (13).

This study presents a comprehensive *in vivo* evaluation of the toxicity associated with scented candle emissions, aiming to address significant gaps in current knowledge. Volatile organic compounds released during candle combustion were identified and characterized using gas chromatography–mass spectrometry (GC–MS), with unscented candles used as baseline controls. Additionally, an in silico ADMET analysis was conducted to predict the toxicity profiles of the detected compounds. Assessment of the biological effects of exposure was performed using healthy adult male Wistar albino rats. Systemic inflammation and oxidative stress were evaluated by measuring serum levels of tumor necrosis factor-alpha (TNF-*α*) and interleukin-6 (IL-6). Lung tissue samples were used for biochemical, molecular, and histological analyses. Malondialdehyde (MDA), catalase (CAT), and total superoxide dismutase (T-SOD) were quantified in tissue homogenates to assess oxidative stress. Additionally, TNF-*α* and cyclooxygenase-2 (COX-2) gene expression levels were measured by real-time quantitative PCR (qRT-PCR), and their localization was examined using immunohistochemistry. Histopathological evaluation was conducted to examine tissue architecture, inflammatory cell infiltration, and structural damage.

## 2 Materials and methods

### 2.1 Animals and experimental design

A total of 54 healthy male Wistar albino rats (8–10 weeks old, 180–200 g) were randomly assigned to nine groups (*n* = 6 per group). Animals were acclimatized for 1 week prior to the experiment and maintained under standard laboratory conditions: temperature (22 ± 2°C), relative humidity (50%), and a 12:12 h light/dark cycle.

The experimental protocol adhered to institutional and international guidelines and was approved by the Institutional Animal Care and Use Committee of Cairo University (CU-IACUC) under approval number CUIF 2825.

- Groups 1–3 were used as control groups and were housed in a non-sealed, well-ventilated room.
- Groups 4–6 were exposed daily to emissions from a single unscented candle for 1, 3, or 6 h, respectively.
- Groups 7–9 were exposed daily to emissions from a single scented candle for 1, 3, or 6 h, respectively.

All exposures were conducted 5 days per week for eight consecutive weeks. The unscented and scented candles used were commercially available products purchased from a major retail store in Tabuk, Saudi Arabia. Exposure sessions were conducted in a sealed, air-conditioned room (3 m × 3 m × 2.8 m), simulating enclosed indoor environments common in the Gulf region. Room temperature (22 ± 2°C) and humidity were continuously monitored and maintained. To ensure consistent exposure, the distance between the burning candle and the rat cages was kept constant across all exposure groups. After each daily exposure session, the animals were returned to the animal facility and maintained under standard laboratory conditions for the remainder of the day.

At the end of the eight-week exposure period, animals were anesthetized, and blood samples were collected via cardiac puncture for serum preparation. The left lung was excised and fixed in 10% neutral buffered formalin for histopathological and immunohistochemical analyses, while the right lung was snap-frozen and stored at −80°C for subsequent biochemical and molecular assessments.

### 2.2 Gas chromatography–mass spectrometry analysis

The sample was extracted by solid phase microextraction (SPME) for 20 min at 50°C and injected into GC. The GC–MS system (Agilent Technologies) was equipped with gas chromatograph (7890B) and mass spectrometer detector (5977A) at Central Laboratories Network, National Research Center, Cairo, Egypt. The GC was equipped with HP-5MS column (30 m x 0.25 mm internal diameter and 0.25 μm film thickness). Analyses were carried out using Hydrogen as the carrier gas at a flow rate of 1.1 mL/min at a splitless injection mode, and the following temperature program: 50°C for 0 min; rising at 5°C /min to 200°C and held for 0 min; rising at 20°C /min to 280°C and held for 6 min. The injector and detector were held at 250°C, 320°C. Mass spectra were obtained by electron ionization (EI) at 70 eV; using a spectral range of m/z 50–600 and solvent delay 0 min. The mass temperature was 230°C and Quad 150°C. Identification of different constituents was determined by comparing the spectrum fragmentation pattern with those stored in Wiley and NIST Mass Spectral Library data.

### 2.3 *In silico* ADMET properties

The determination of ADMET properties is essential not only in drug discovery but also in evaluating the potential health risks associated with environmental exposures. In this study, we assessed the ADMET profiles of VOCs emitted from both unscented and scented candles. The chemical structures of the VOCs were drawn and converted into SMILES format using ChemDraw Ultra, then submitted to the ADMET lab 2.0 online prediction platform. This in silico analysis provided insights into the physicochemical properties and potential biological effects of the VOCs, thereby contributing to the toxicological evaluation of candle emissions.

### 2.4 Assessment of inflammatory and oxidative stress markers

Systemic inflammatory markers were measured in serum, while oxidative stress markers were evaluated in lung tissue homogenates. Twenty milligrams of lung tissue was finely chopped using scissors and placed into a homogenization tube containing three steel beads. Phosphate-buffered saline (PBS) was added at a 1:9 (w/v) ratio, and the mixture was thoroughly homogenized. The homogenate was then centrifuged at 10,000 rpm for 10 min at 4°C, and the resulting supernatant was collected for analysis. The levels of oxidative stress markers were quantified according to the manufacturer’s instructions.

Details of the assay kits used are provided in Table 1.

**Table 1:** List of assay kits utilized for biochemical analyses.

Markers	Elabscience catalog number	Category	Assay type	Sample type
TNF-α	E-EL-R2856	Inflammatory marker	ELISA	Serum
IL-6	E-EL-R0015			
MDA	E-BC-K025-M	Oxidative stress marker	Colorimetric	Lung tissue homogenate
CAT	E-BC-K031-M			
T-SOD	E-BC-K019-M			

### 2.5 mRNA expression levels of TNF-*α* and COX-2 determined by qRT-PCR

Total RNA was isolated from lung tissues using QIAzol reagent (Qiagen, Germany) in accordance with the manufacturer’s protocol. The absorbance ratios at 260/280 and 260/230 nm were measured using a NanoDrop 2000 spectrophotometer (Thermo Scientific, United States) to assess the purity and concentration of the RNA samples. Complementary DNA (cDNA) was synthesized from total RNA using the High-Capacity cDNA Reverse Transcription Kit (Thermo Fisher Scientific, United States). Quantitative PCR (qPCR) was carried out using SYBR Green Master Mix (Applied Biosystems, Life Technologies, United States). Each reaction was performed in triplicate, and target gene expression levels were normalized to *β*-actin as the reference gene. The primer sequences used in this study are listed in Table 2.

**Table 2:** Primer sequences utilized in qRT-PCR.

Target gene	Forward sequence (5′ − 3′)	Reverse sequence (5′ − 3′)	Accession no.	Ref.
TNF-α	TGGGCTCCCTCTCATCAGTTC	TCCGCTTGGTGGTTTGCTAC	NM_012675.3	(43)
COX − 2	AGAAGCGAGGACCTGGGTTCAC	ACACCTCTCCACCGATGACCTG	NM_017232.4	(44)
β-Actin	TCCCTGGAGAAGAGCTATGA	ATAGAGCCACCAATCCACAC	NM_031144.3	(44)

### 2.6 Immunohistochemistry of lung tissue

Paraffin sections were mounted on positively charged slides by using avidinbiotin- peroxidase complex (ABC) method. Primary antibodies against (TNF-*α* & COX-2) were used for detection (Table 3). Sections from each group were incubated with these antibodies, then the reagents required for ABC method were added (Vectastain ABC-HRP kit, Vector laboratories). Marker expression was labeled with peroxidase and colored with diaminobenzidine (DAB, produced by Sigma) to detect antigen–antibody complex. Negative controls were included using non-immune serum in place of the primary or secondary antibodies. IHC stained sections were examined via using Olympus microscope (BX-63) (14).

**Table 3:** Antibodies utilized in immunohistochemical study.

Antibody	Company name	Type	Host	Catalog No.	dilution
TNF-α	Service bio	Polyclonal	Rabbit	GB11188	1:500
COX-2	Novusbio	Polyclonal	Rabbit	NB100-689	1:100

The Scoring of immunohistochemistry results by determination of reaction area percent in 10 microscopic fields using image J 1.53 t, Wayne Rasband and contributors, National Institutes of Health, United States.

### 2.7 Histological examination of lung tissue

Specimens were fixed in 10% neutral buffer formalin, then trimmed, washed in water, dehydrated in ascending grades of ethyl alcohol, cleared in xylene and embedded in paraffin. Thin sections (4-6 *μ*) were processed and stained with Hematoxylin and Eosin stain (15). Histopathological alterations were graded semiquantitatively on a four-point scale: absent (−), mild (+), moderate (++), and severe (+++).

### 2.8 Statistical analysis

Statistical analyses were performed using GraphPad Prism 9.5.1 (GraphPad Software Inc., San Diego, CA, United States). Raw data were statistically analyzed for normal distribution using the Shapiro–Wilk test. The data were expressed as the mean ± SD (*n* = 6). Statistical analysis was performed using two-way analysis of variance (ANOVA) to assess the effects of time (1 h/day, 3 h/day, and 6 h/day) and treated groups (control, unscented candle, and scented candle groups) and their interaction on the measured parameters. *Post hoc* comparisons were conducted using Tukey’s test. A *p*-value < 0.05 was considered statistically significant. ns: non-significant; *: significant at *p* < 0.05 level; **: significant at *p* < 0.01 level; ***: significant at *p* < 0.001 level; and****: significant at *p* < 0.0001 level. The number of rats used per group was calculated using GPower 3.1.9.4 (Heinrich Heine University Düsseldorf, Düsseldorf, Germany) (16). Taking into consideration our preliminary results, we have considered an effect size of 1.1. Determining, based on significance level (*α*) at 0.05 and testing power (1-*β*) at 0.8 for 9 groups, a total of 54 adult male Wistar rats was determined (6 rats/group).

## 3 Results

### 3.1 GC–MS analysis

**Figure 1:**
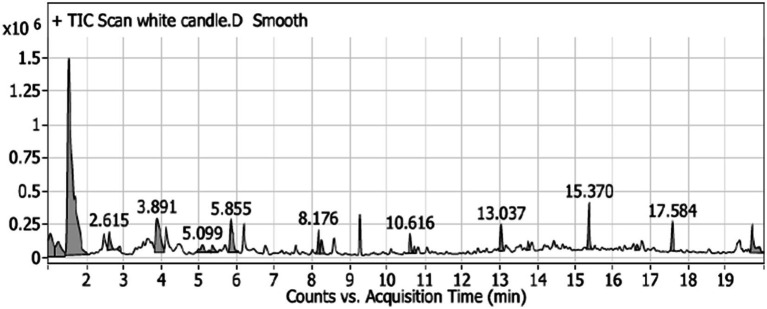
GC–MS chromatogram of the unscented (white) candle sample, showing the detected VOCs over the retention time. Major peaks are labeled with their corresponding retention times (in minutes).

The GC–MS analysis of the unscented candle revealed the presence of 20 VOCs, as presented in Table 4 and illustrated in Figure 1. The identified compounds primarily include hydrocarbons, aldehydes, ketones, and carboxylic acids, with toluene being the most abundant, accounting for approximately 51.65% of the total VOCs profile.

**Table 4:** Volatile organic compounds identified in the unscented candle sample by GC–MS analysis, including retention time (RT), compound name, molecular formula, peak area, and relative abundance (area %).

Peak	RT	Name	Formula	Area	Area Sum %
1	1.051	Butane, 2-isothiocyanato-	C5H9NS	1742912.3	6.51
2	1.258	1,4,2,5 Cyclohexanetetrol	C6H12O4	1174054.9	4.38
3	1.55	Toluene	C7H8	13,833,310	51.65
4	2.615	Heptanal	C7H14O	619533.11	2.31
5	2.89	Hexanoic acid	C6H12O2	129238.27	0.48
6	3.891	Decane	C10H22	2081797.8	7.77
7	5.099	trans-p-mentha-1(7),8-dien-2-ol	C10H16O	373396.09	1.39
8	5.267	7-Hexadecenal, (Z)-	C16H30O	49350.37	0.18
9	5.368	2(3H)-Furanone, 5-heptyldihydro-	C11H20O2	231381.07	0.86
10	5.855	Undecane	C11H24	1260119.9	4.71
11	8.176	Dodecane	C12H26	543196.39	2.03
12	8.258	2-Decanone	C10H20O	360386.48	1.35
13	10.616	Tridecane	C13H28	420609.45	1.57
14	10.722	2-Undecanone	C11H22O	174220.63	0.65
15	13.037	Tetradecane	C14H30	576324.56	2.15
16	13.75	Naphthalene, 1,7-dimethyl-	C12H12	207105.1	0.77
17	15.37	Pentadecane	C15H32	1056270.8	3.94
18	16.646	3-(2-Methyl-propenyl)-1H-indene	C13H14	129218.31	0.48
19	17.584	Hexadecane	C16H34	613648.79	2.29
20	19.705	Heptadecane	C17H36	1204849.9	4.5

**Figure 2:**
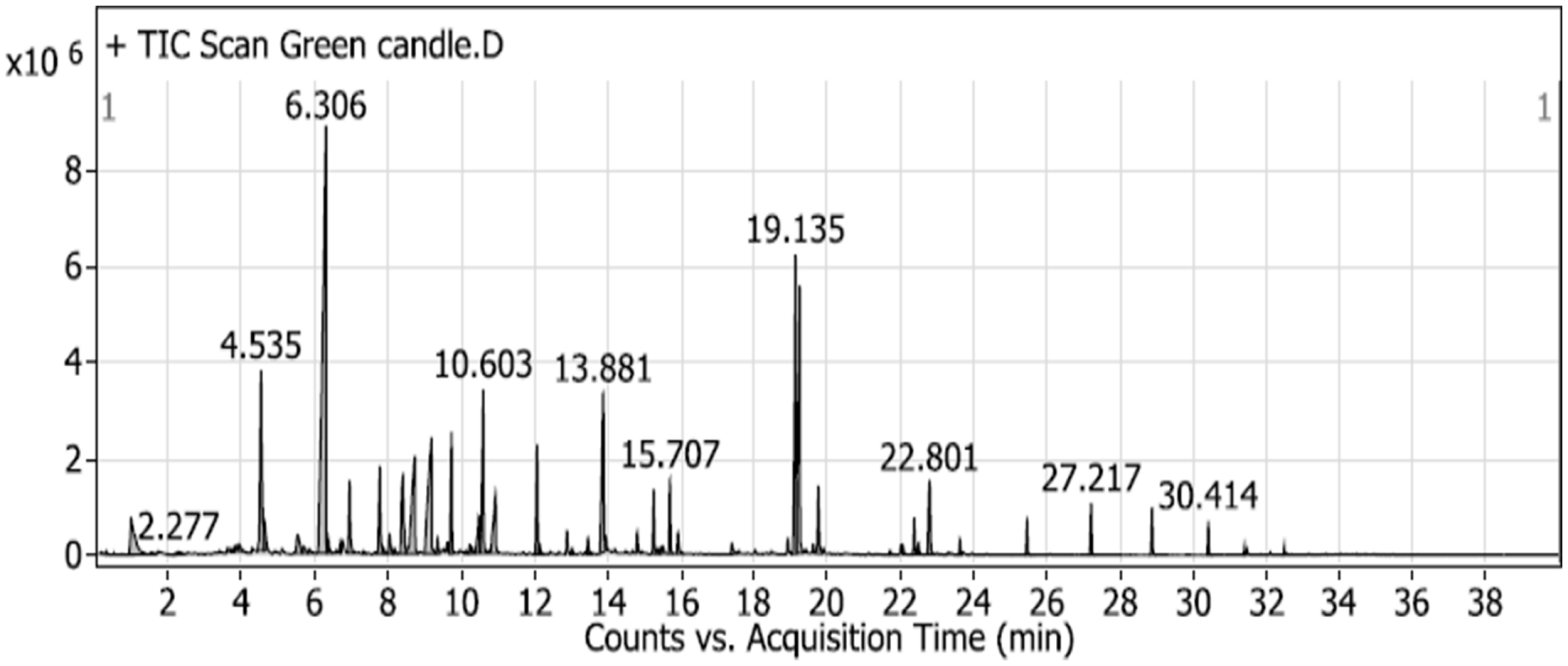
GC–MS chromatogram of unscented candle (green candle) sample, illustrating the VOCs emitted over the acquisition time. Major peaks are labeled with their respective retention times (in minutes).

The GC–MS analysis of the scented candle revealed the presence of 60 VOCs, as listed in Table 5 and illustrated in Figure 2. These compounds encompass a broad range of chemical classes, including terpenes, esters, alcohols, aldehydes, and aromatic hydrocarbons. Notably, linalool was the most abundant compound, comprising 22.48% of the total VOCs profile, followed by cyclopentaneacetic acid, 3-oxo-2-pentyl- methyl ester (8.04%) and amberonne (7.03%) and methylene chloride (2.71%).

**Table 5:** Volatile organic compounds identified in the scented (green) candle sample by GC–MS analysis, including retention time (RT), compound name, molecular formula, peak area, and relative abundance (area %).

Peak	RT	Name	Formula	Area	Area Sum %
1	0.982	Methylene chloride	CH2Cl2	6694931.95	2.71
2	1.526	1,3,5-Cycloheptatriene	C7H8	200548.27	0.08
3	2.277	Bicyclo [2.1.1] hexan-2-ol, 2-ethenyl-	C8H12O	482942.24	0.2
4	3.691	(3-Amino-4,6-dimethylthieno[2,3-b] pyridin-2-yl) (phenyl)methanone	C16H14N2OS	285283.05	0.12
5	3.803	Bicyclo [3.1.1] heptane, 6,6-dimethyl-2-methylene-, (1S)-	C10H16	309723.84	0.13
6	3.935	Cyclohexene, 1-(2-nitro-2-propenyl)-	C9H13NO2	614687.05	0.25
7	4.535	D-Limonene	C10H16	13,836,898	5.61
8	4.635	Eucalyptol	C10H18O	1838070.07	0.74
9	5.536	7-Octen-2-ol, 2,6-dimethyl-	C10H20O	2295753.9	0.93
10	5.699	Hexane, 1-chloro-5-methyl-	C7H15Cl	746824.35	0.3
11	5.855	Isopulegol	C10H18O	376313.25	0.15
12	6.306	Linalool	C10H18O	55492727.8	22.48
13	6.381	2H-Pyran, tetrahydro-4-methyl-2-(2-methyl-1-propenyl)-	C10H18O	710211.3	0.29
14	6.712	Phenylethyl Alcohol	C8H10O	606846.74	0.25
15	6.956	1,2-Dihydrolinalool	C10H20O	4148345.95	1.68
16	7.332	Cyclohexanol, 2-methyl-5-(1-methylethenyl)-, (1.alpha.,2.beta.,5.alpha.)-	C10H18O	241875.24	0.1
17	7.782	Acetic acid, phenylmethyl ester	C9H10O2	5345460.1	2.17
18	7.888	endo-Borneol	C10H18O	361033.18	0.15
19	8.045	cis-Ethyl-linalyl acetate	C13H22O2	1384508.74	0.56
20	8.182	Dodecane	C12H26	309702.57	0.13
21	8.414	p-Methylbenzyl acetate	C10H12O2	7150628.69	2.9
22	8.739	Citronellal	C10H18O	12803675.9	5.19
23	9.189	Carbonic acid, but-3-yn-1-yl octyl ester	C13H22O3	15697293.2	6.36
24	9.358	2-Octen-1-ol, 3,7-dimethyl-	C10H20O	805967.38	0.33
25	9.74	Linalyl acetate	C12H20O2	5942605.34	2.41
26	10.146	2,4-Heptadienal, 2,4-dimethyl-	C9H14O	119088.66	0.05
27	10.234	Cyclohexanemethanol, 4-(1-methylethyl)-, trans-	C10H20O	426323.8	0.17
28	10.297	Cyclohexanemethanol, 4-(1-methylethyl)-, cis-	C10H20O	443133.56	0.18
29	10.478	(1S,3S,4S,5R)-1-Isopropyl-4-methylbicyclo [3.1.0] hexan-3-ol	C10H18O	2691527.87	1.09
30	10.603	Bicyclo [2.2.1] heptan-2-ol, 1,7,7-trimethyl-, acetate, (1S-endo)-	C12H20O2	9324310.66	3.78
31	10.941	Octanal, 7-hydroxy-3,7-dimethyl-	C10H20O2	5943565.9	2.41
32	12.079	ALPHA.-TERPINENYL ACETATE	C12H20O2	5837071.17	2.36
33	12.148	Bicyclo[4.1.0] heptane, 3,7,7-trimethyl-, [1S-(1.alpha.,3.beta.,6.alpha.)]-	C10H18	572319.6	0.23
34	13.036	Limonen-6-ol, pivalate	C15H24O2	389401.81	0.16
35	13.468	Diphenyl ether	C12H10O	803971.26	0.33
36	13.549	Caryophyllene	C15H24	118914.19	0.05
37	13.881	exo-7-(2-Propenyl) bicyclo[4.2.0]oct-1(2)-ene	C11H16	12607232.1	5.11
38	13.962	.beta.-Guaiene	C15H24	443168.73	0.18
39	14.813	3-(4-Isopropylphenyl)-2-methylpropionaldehyde	C13H18O	1052414.94	0.43
40	15.263	3-Buten-2-one, 4-(2,6,6-trimethyl-1-cyclohexen-1-yl)-	C13H20O	2999061.25	1.22
41	15.37	Retinal	C20H28O	291444.08	0.12
42	15.464	Benzenepropanal, 4-(1,1-dimethylethyl)-	C13H18O	377535.88	0.15
43	15.707	Butylated Hydroxytoluene	C15H24O	3220204.06	1.3
44	15.926	3-tert-Butyl-4-hydroxyanisole	C11H16O2	1343565.39	0.54
45	17.396	2-Methoxy-4-methyl-1-pentylbenzene	C13H20O	876447.42	0.36
46	19.135	Amberonne (isomer 1)	C16H26O	17342411.4	7.03
47	19.248	Cyclopentaneacetic acid, 3-oxo-2-pentyl-, methyl ester	C13H22O3	19851069.5	8.04
48	19.761	Amberonne (isomer 3)	C16H26O	3913438.72	1.59
49	21.719	Octadecane	C18H38	268347.48	0.11
50	22.069	Naphthalene, 6,7-diethyl-1,2,3,4-tetrahydro-1,1,4,4-tetramethyl-	C18H28	441420.01	0.18
51	22.382	Isopropyl myristate	C17H34O2	1676524.48	0.68
52	22.501	Cyclopentadecanone, 2-hydroxy-	C15H28O2	598947.58	0.24
53	22.801	Cyclopenta[g]-2-benzopyran, 1,3,4,6,7,8-hexahydro-4,6,6,7,8,8-hexamethyl-	C18H26O	5523927.47	2.24
54	23.627	Nonadecane	C19H40	686739.07	0.28
55	25.466	Eicosane	C20H42	1624424.53	0.66
56	27.217	Heneicosane	C21H44	2437512.36	0.99
57	28.881	Docosane	C22H46	2152875.19	0.87
58	30.414	Tricosane	C23H48	1225695.28	0.5
59	31.402	Tetracosane	C24H50	264062.15	0.11
60	32.491	Bis(2-ethylhexyl) phthalate	C24H38O4	254810.51	0.1

### 3.2 Physicochemical and ADMET evaluation of VOCs

Although Supplementary Table S1 includes the complete list of VOCs emitted from unscented candles, the results presented here emphasize compounds with the most significant toxicological profiles. These compounds exhibited high lipophilicity, e.g., Decane LogP: 6.66; Tetradecane LogP: 7.565 and low aqueous solubility (e.g., Hexadecane LogS: −7.084), consistent with their volatile nature. Several compounds showed high predicted BBB permeability (e.g., Heptanal, 2-Decanone), while others demonstrated rapid clearance (e.g., Toluene CL: 10.443 L/h/kg) and short half-lives (e.g., Heptadecane T½: 0.058 h).

Naphthalene, 1,7-dimethyl- was flagged for high DILI risk, Ames positivity, and genotoxicity alerts. Aldehydes (e.g., Heptanal, 7-Hexadecenal) and Butane, 2-isothiocyanato- exhibited structural alerts (Alarm_NMR, BMS) and potential for respiratory and skin sensitization. Hydrocarbons such as Decane and Undecane showed low predicted toxicity and favorable metabolic stability. Some compounds, including Decane (BCF: 2.838), indicated potential for bioaccumulation. Notably, Butane, 2-isothiocyanato- and 3-(2-Methyl-propenyl)-1H-indene demonstrated high Tox21 activity, indicating potential biological reactivity.

Although Supplementary Table S2 presents the complete ADMET profiles of all VOCs emitted from scented candles, the discussion here focuses on the compounds with the most concerning toxicological properties. These compounds generally exhibited high lipophilicity and low QED values. Several showed high predicted BBB permeability, including Methylene chloride, Bicyclo [2.1.1] hexan-2-ol, and 2-ethenyl-. Most compounds had short, predicted half-lives and rapid clearance. Retinal showed slow clearance and high Tox21 activity. Aldehyde-containing compounds (e.g., Citronellal, 2,4-Heptadienal) triggered reactivity alerts (Alarm_NMR, BMS). Terpenoids (e.g., D-Limonene, Eucalyptol) showed low toxicity and good metabolic stability. In contrast, [3-Amino-4,6-dimethylthieno (2,3-b) pyridin-2-yl] (phenyl) methanone was associated with high CYP inhibition and Tox21 activity.

### 3.3 Measurement of serum inflammatory markers TNF-*α* and IL-6

**Figure 3:**
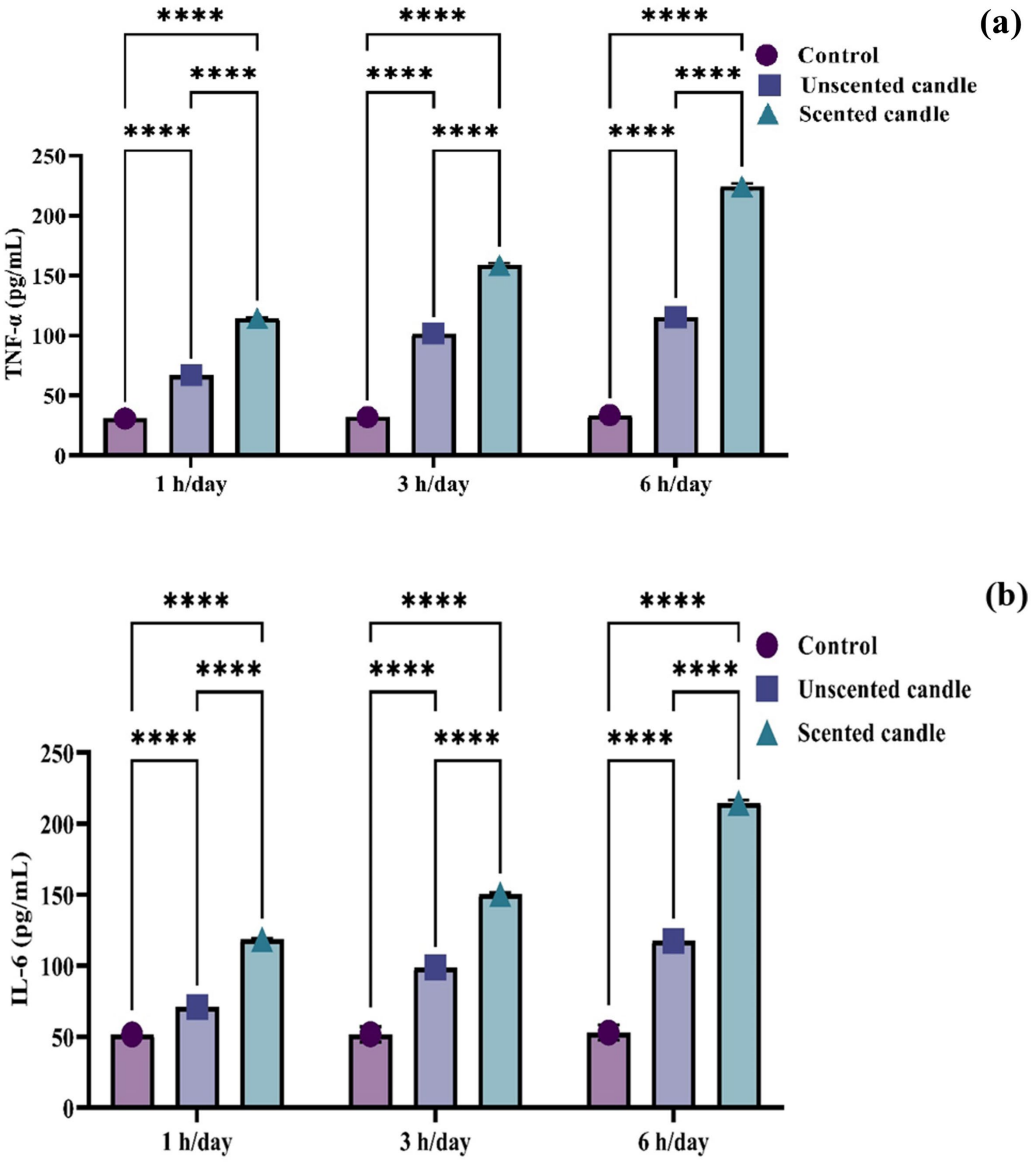
ELISA-based quantification of serum proinflammatory cytokines: **(a)** TNF-*α* and **(b)** IL-6. Data are presented as mean ± SD (*n* = 6 per group). Statistical differences were considered significant at *p* < 0.05.

To evaluate systemic inflammation, serum levels of the pro-inflammatory cytokines TNF-α and IL-6 were quantified using ELISA. As shown in Figures 3a,b, both cytokines exhibited a statistically significant, exposure duration-dependent increase in response to emissions from both unscented and scented candles (*p* < 0.05). At each exposure duration (1 h, 3 h, and 6 h), TNF-α and IL-6 levels were consistently higher in the scented candle group compared to the unscented group. The increases became more pronounced with longer daily exposures. In contrast, the control group, which was exposed only to fresh air, showed no significant change in cytokine levels across the study period.

### 3.4 Assessment of oxidative stress biomarkers in lung tissue via colorimetric analysis

**Figure 4:**
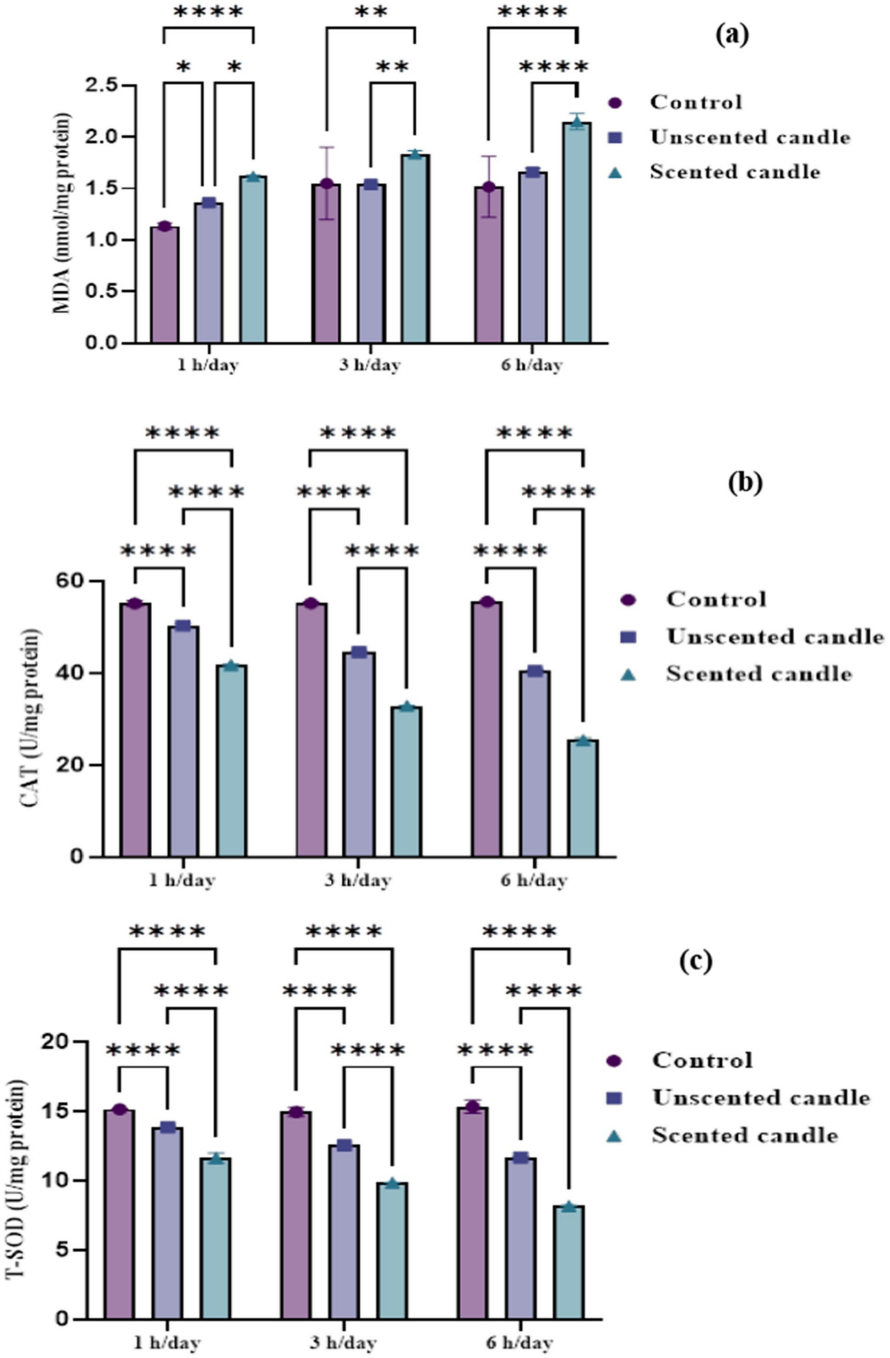
Oxidative stress biomarkers in lung tissue homogenates: **(a)** MDA, **(b)** CAT, and **(c)** T-SOD. Data are expressed as mean ± SD (*n* = 6). Statistical significance was set at *p* < 0.05.

Malondialdehyde levels in lung tissue, shown in Figure 4a, increased significantly in a duration-dependent manner following exposure to both unscented and scented candle emissions (*p* < 0.05). At all exposure durations (1 h, 3 h, and 6 h/day), MDA levels were significantly higher in the exposed groups compared to the control, with the highest concentrations observed in the scented candle group. No significant changes in MDA levels were observed in the control group throughout the study period.

Catalase and total superoxide dismutase activities, shown in Figures 4b,c, respectively, declined significantly with increasing exposure duration. A mild reduction in both enzyme activities was noted after short-term exposure (1 h/day), with a more pronounced decline in the scented candle group. As exposure duration increased to 3 and 6 h/day, enzymatic activity decreased progressively in both exposure groups, with the scented candle group consistently showing a greater reduction at each time point. By 6 h/day, CAT and T-SOD levels were markedly suppressed in the scented group. In contrast, the control group maintained stable antioxidant enzyme activity, with no significant changes observed.

### 3.5 The expression levels of TNF-*α* and COX-2

**Figure 5:**
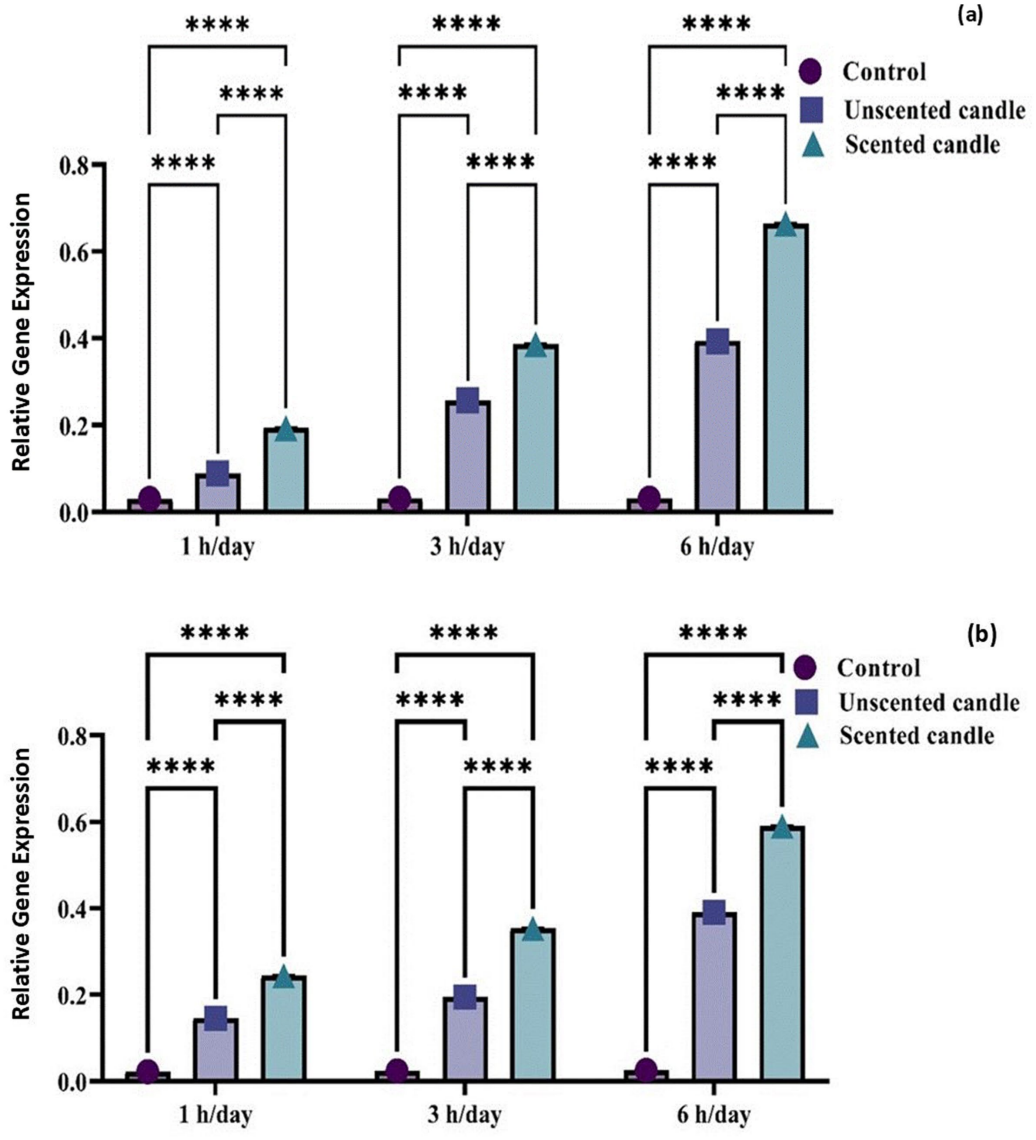
Relative gene expression of TNF-α **(a)** and COX-2 **(b)** in control and candle-exposed groups. Data are presented as mean ± SD. Statistical analysis was performed using [ANOVA/*t*-test, specify if relevant], with significance set at *p* < 0.05.

Exposure to emissions from both unscented and scented candles resulted in a significant increase in TNF-*α* and COX-2 mRNA expression levels compared to the control group at all exposure durations (1, 3, and 6 h/day), as illustrated in Figures 5a,b. The differences among groups were highly significant at each time point (*p* < 0.0001). An exposure duration-dependent trend was evident, with expression levels increasing progressively with longer exposure. Notably, the scented candle group exhibited significantly higher expression levels than the unscented candle group, indicating a more pronounced pro-inflammatory response.

### 3.6 Immunohistochemistry examination

**Figure 6:**
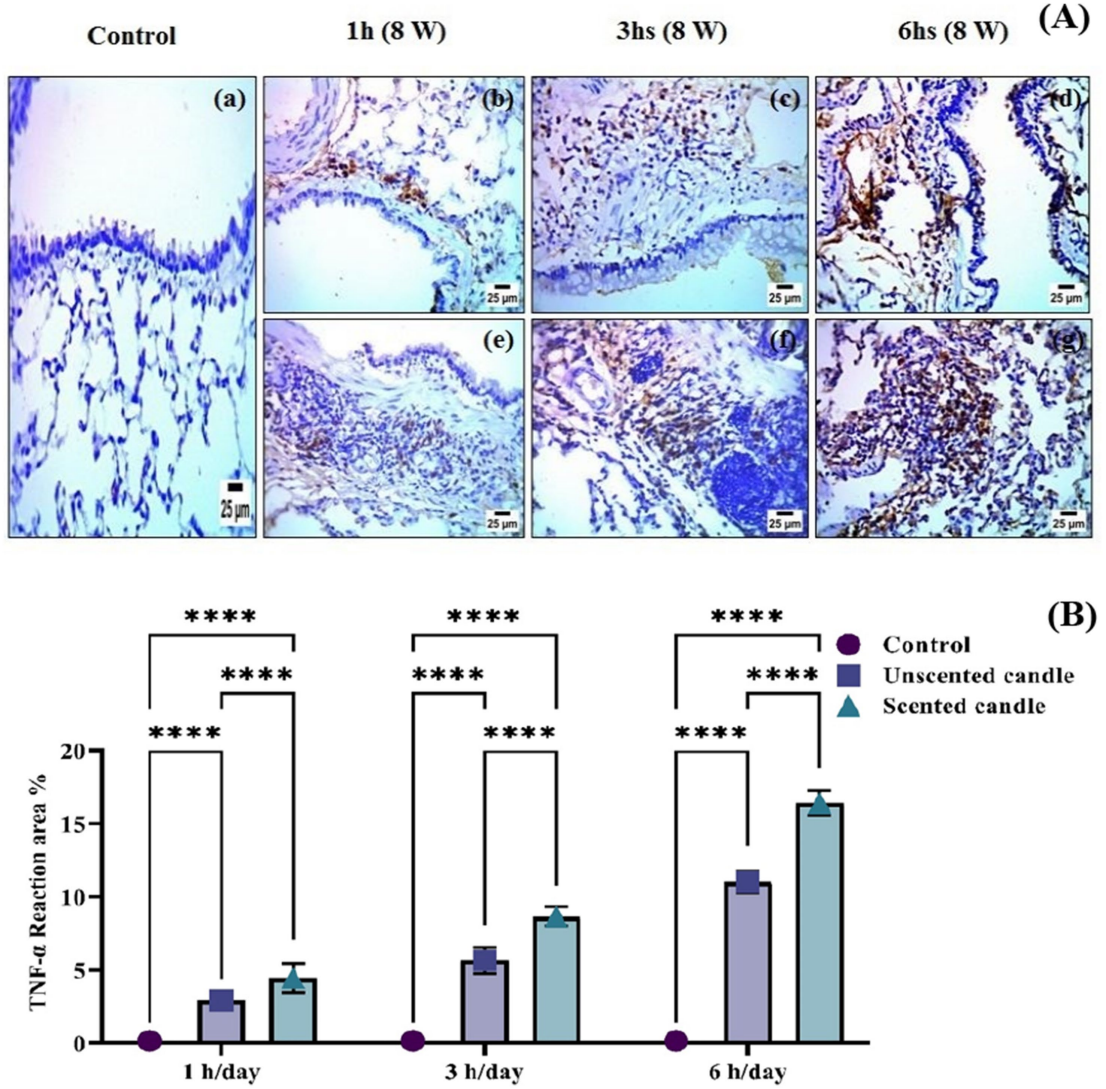
**(A)** Immunohistochemical staining of TNF-α in lung tissue sections: **(a)** control; **(b–d)** exposure to unscented candles for 1 h, 3 h, and 6 h, respectively; **(e–g)** exposure to scented candles for 1 h, 3 h, and 6 h, respectively. **(B)** The Scoring of immunohistochemistry results. Data are expressed as mean ± SD. Statistical differences were considered significant at *p* < 0.05.

**Figure 7:**
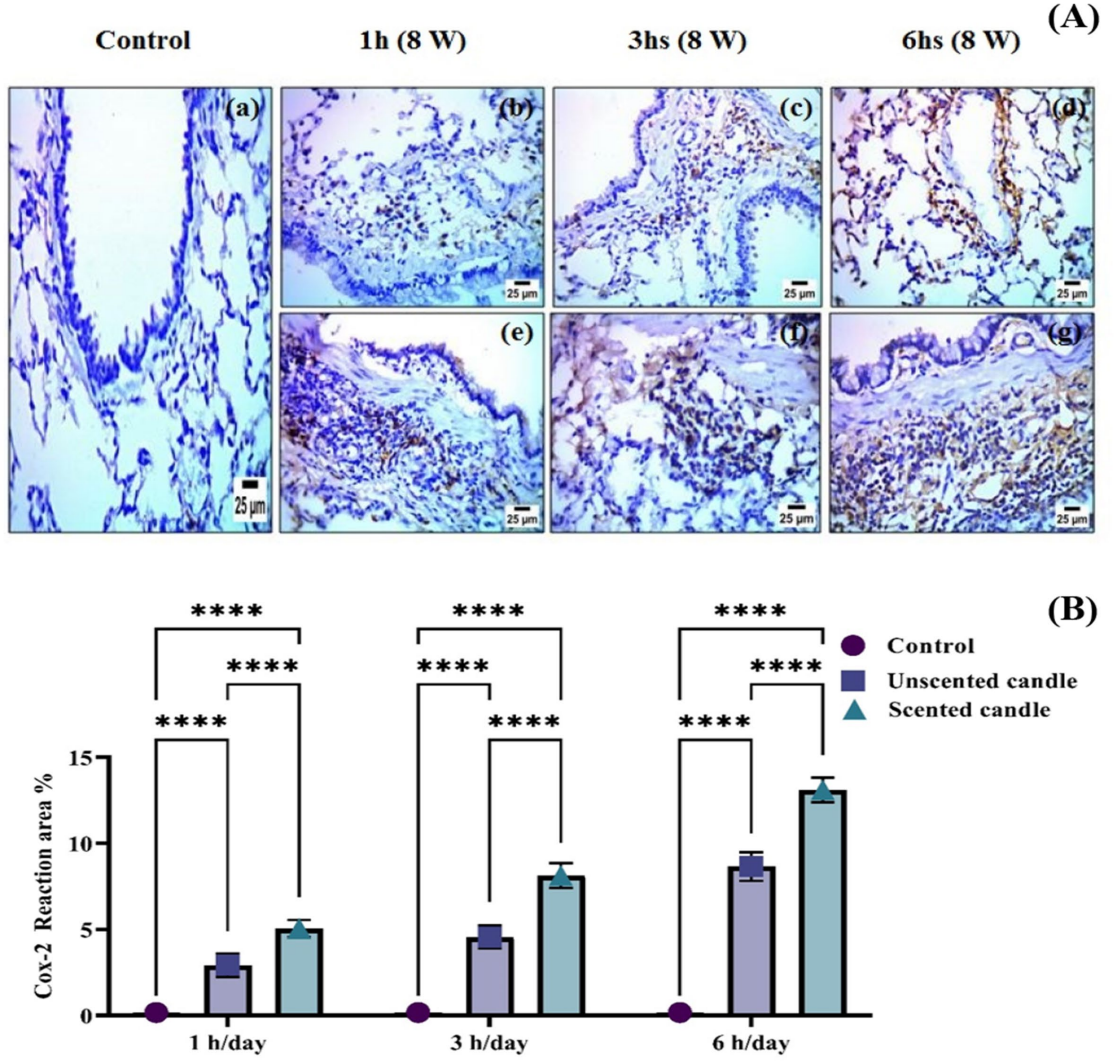
**(A)** Immunohistochemical staining of COX-2 in lung tissue sections: **(a)** control; **(b–d)** exposure to unscented candles for 1 h, 3 h, and 6 h, respectively; **(e–g)** exposure to scented candles for 1 h, 3 h, and 6 h, respectively. **(B)** The Scoring of immunohistochemistry results. Data are expressed as mean ± SD. Statistical differences were considered significant at *p* < 0.05.

Immunohistochemical analysis revealed a significant increase in TNF-*α* and COX-2 protein expression in the lung tissues of rats exposed to emissions from both unscented and scented candles, compared to the control group. As shown in Figure 6A, immunohistochemical staining for TNF-*α* was nearly absent in the control group (panel a), while exposure to unscented candles for 1, 3, and 6 h (panels b–d) and scented candles (panels e–g) resulted in progressively increased staining intensity, reflecting elevated TNF-*α* expression. Similarly, Figure 7A shows COX-2 staining patterns, with minimal immunoreactivity in the control (panel a) and increased expression in the unscented (panels b–d) and scented (panels e–g) candle exposure groups in a duration-dependent manner.

These observations were further confirmed by quantitative immunostaining scores presented in Figures 6B, 7B for TNF-α and COX-2, respectively. Statistical analysis showed highly significant differences (**p < 0.0001) between exposed groups and controls across all exposure durations. Additionally, exposure to scented candles consistently resulted in higher expression levels of both TNF-α and COX-2 compared to unscented candles, indicating a more intense pro-inflammatory response associated with scented candle emissions.

### 3.7 Histopathological examination

**Figure 8:**
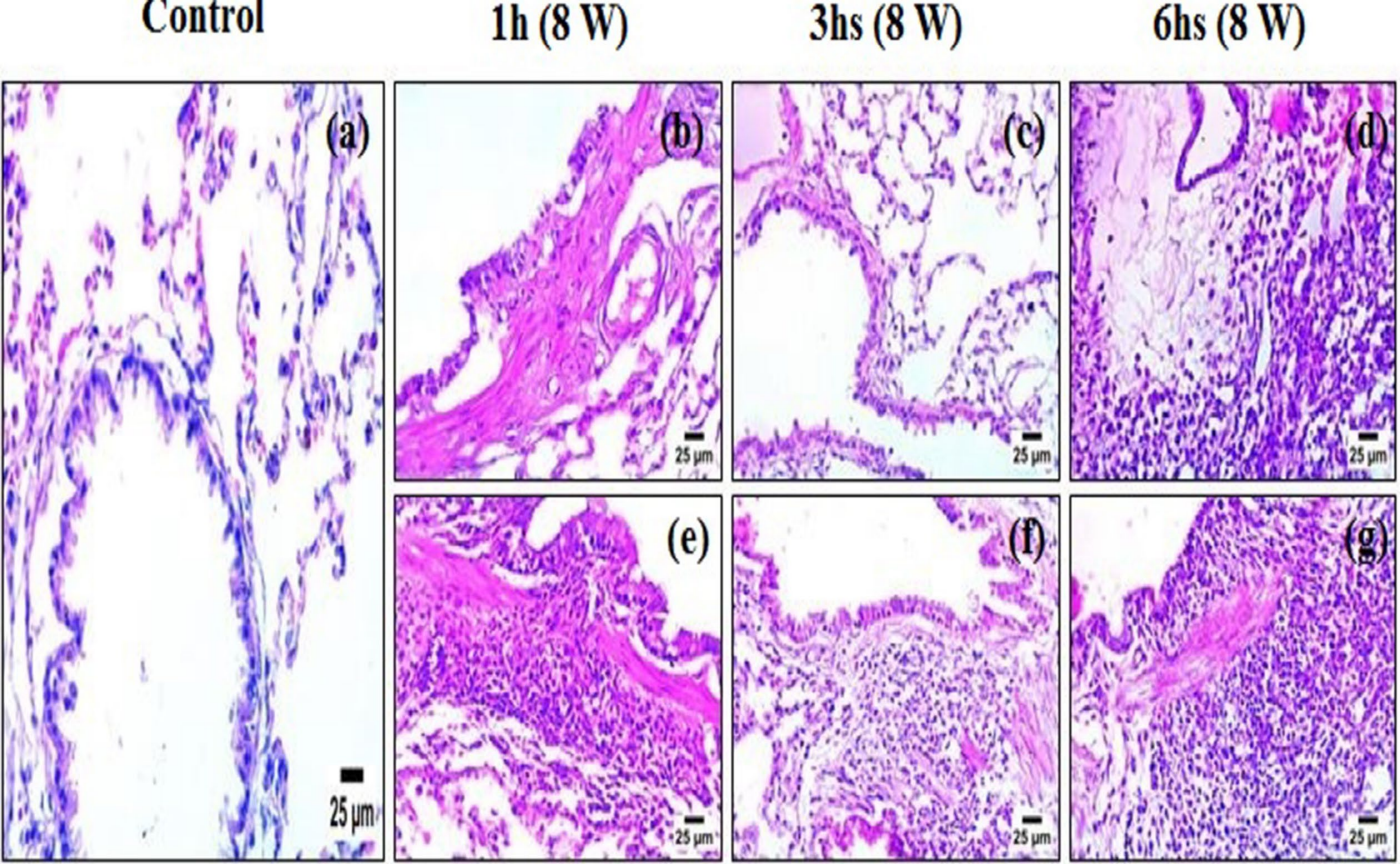
Photomicrographs of lung tissue sections stained with hematoxylin and eosin (H&E): **(a)** control; **(b–d)** exposure to unscented candles for 1 h, 3 h, and 6 h, respectively; **(e–g)** exposure to scented candles for 1 h, 3 h, and 6 h, respectively.

Histological analysis of lung tissues (Figures 8a–g) revealed a progressive pattern of structural damage that correlated with both the duration and type of candle exposure. In the control group (Figure 8a), the lung architecture appeared normal, with well-preserved alveolar spaces, bronchioles, and no pathological alterations observed. In rats exposed to unscented candle emissions (Figures 8b–d), changes progressed in a time-dependent manner: mild inflammatory infiltration and peribronchiolar fibrosis after 1-h, moderate interstitial thickening and fibrotic remodeling after 3 h, and severe diffuse disruption with dense inflammatory cell accumulation after 6 h. More pronounced effects were observed in the scented candle groups (Figures 8e–g), where moderate epithelial degeneration and interstitial alterations were already evident after 1 h, advancing to severe necrobiotic degeneration, fibrotic remodeling, and dense inflammatory infiltration after 3 h, and persisting as severe injury at 6 h (Table 6).

**Table 6:** Semiquantitative histopathological scores corresponding to Figure 8.

Figure panel	(a)	(b)	(c)	(d)	(e)	(f)	(g)
Score	−	+	++	+++	++	+++	+++

Lesions were graded on a four-point scale: absent (−), mild (+), moderate (++), and severe (+++).

## 4 Discussion

### 4.1 Implications of GC–MS and ADMET profiling

Scented candles emit VOCs during combustion, many of which have been associated with respiratory irritation, oxidative stress, and other potential health hazards (11). In the present study, GC–MS analysis revealed that the unscented candle, although originally intended as a negative control, emitted a complex mixture of VOCs, including several compounds with potential toxicological relevance. In silico ADMET profiling further indicated that compounds detected in both candle groups possessed physicochemical properties consistent with health risk, such as high lipophilicity and poor aqueous solubility.

Specifically, in the unscented candle emissions, hydrocarbons such as decane and tetradecane were predominant and exhibited high lipophilicity and low water solubility—characteristics linked to increased volatility, enhanced membrane permeability, and the potential for bioaccumulation. These findings suggest that despite clear differences in VOC composition, emissions from both scented and unscented candles may contribute to adverse health effects (17).

Notably, decane also showed a measurable bioaccumulation factor, indicating its potential to persist in lipid-rich tissues and contribute to chronic exposure risks, particularly with repeated or long-term inhalation. Furthermore, the low aqueous solubility of many identified compounds—such as hexadecane—suggests that, once deposited in biological systems, their elimination via aqueous-phase excretion (e.g., renal clearance) may be limited. This physicochemical property is known to increase the risk of bioaccumulation, as reported in earlier studies (18). Evidence from human and experimental studies indicates that compounds such as decane, tetradecane, and hexadecane—identified in the present analysis—can cause sensory irritation and skin toxicity, reinforcing concerns about their potential health effects even at low environmental concentrations (19).

Of particular concern are compounds with predicted BBB permeability, such as heptanal and 2-decanone, which may cross into the central nervous system (CNS) and exert neurotoxic effects. This aligns with previous studies indicating that airborne aldehydes and ketones can cause CNS depression and cognitive impairment at sufficient concentrations (20). Toluene, the predominant VOC in emissions from the unscented candle (51.65%), is known for its high absorption rate via inhalation—the most common route of exposure in both occupational and environmental settings (21). Although ADMET predictions indicated a high clearance rate, suggesting rapid systemic elimination, repeated inhalation may still result in tissue accumulation and associated toxicity. Due to its high lipophilicity, toluene readily crosses biological membranes, including the blood–brain and placental barriers. Once absorbed, it distributes widely throughout the body, with preferential accumulation in highly perfused organs such as the brain, liver, kidneys, and bone marrow. Systemic peak concentrations are typically reached within 15 to 30 min following inhalation (22). Several compounds identified in the emissions raise additional toxicological concerns. Naphthalene, 1,7-dimethyl-, though present at a low concentration (0.77%), has been linked to drug-induced liver injury, genotoxicity, and respiratory tract tumors in rodents (23). Butane, 2-isothiocyanato- (6.51%), exhibited strong Tox21 activity and structural alerts for respiratory and dermal sensitization, and is classified as acutely toxic (24).

GC–MS analysis of scented candle emissions revealed a diverse and chemically complex profile, with 60 volatile compounds identified per candle. Consistent with these findings, in-silico ADMET evaluations flagged several compounds as potentially hazardous, highlighting their capacity for bioaccumulation, membrane permeability, or organ-specific toxicity. Among these, methylene chloride was identified and is recognized for its acute toxicity and carcinogenic potential (25, 26). The metabolic fate of methylene chloride in rats has been explored in earlier studies, which describe two primary biotransformation pathways. The first involves a mixed-function oxidase system linked to cytochrome P450 enzymes in the mitochondria, leading to the production of carbon monoxide as the main end product (27). The second is a glutathione-dependent pathway, mediated by glutathione-S-transferase in the cytoplasm, resulting in the formation of formaldehyde, which is subsequently oxidized to carbon dioxide *in vivo* (28).

Several aldehydes, such as citronellal and 2,4-heptadienal, triggered structural alerts in silico, suggesting potential for protein binding and sensitization due to their chemical reactivity—an interpretation supported by previous studies reporting similar effects for reactive aldehydes (29, 30). Additionally, (3-amino-4,6-dimethylthieno [2,3-b] pyridin-2-yl)(phenyl) methanone showed strong CYP inhibition and Tox21 activity, suggesting possible metabolic toxicity despite its low concentration (0.12%) (31). Overall, the detection of multiple toxic and bioactive VOCs in both unscented and scented candle emissions highlights their potential to impair indoor air quality and contribute to cumulative health risks upon repeated exposure.

### 4.2 Pro-inflammatory response induced by candle emissions

The biological significance of these findings was evidenced by the marked elevation of serum TNF-*α* and IL-6 levels in the groups exposed to candle emissions, indicating a strong pro-inflammatory response. Importantly, exposure to scented candles resulted in significantly higher cytokine concentrations compared to unscented candles, with the highest TNF-α levels observed after 6 h of daily exposure. These levels were substantially higher than those observed in both the unscented candle and control groups, suggesting that the emission of over 60 volatile compounds from a single scented candle may significantly amplify inflammatory effects. Previous studies have shown that air pollution can trigger immune activation and excessive cytokine production (12, 32). Additionally, other reports have documented that exposure to air pollution can lead to a wide spectrum of short- and long-term health effects across multiple body systems. These include mild irritation of the upper respiratory tract, the development or exacerbation of chronic respiratory and cardiovascular conditions, respiratory infections in children, chronic bronchitis in adults, lung cancer, and the worsening of pre-existing diseases such as asthma (33).

### 4.3 Impact of candle-derived VOCs on pulmonary oxidative stress

The current study demonstrated a significant oxidative imbalance in lung tissues of rats exposed to both unscented and scented candle emissions, as indicated by elevated MDA levels and reduced activities of CAT and T-SOD. MDA, a widely recognized marker of lipid peroxidation, increased significantly in a time-dependent manner, with the most pronounced effect observed in the scented candle group. This elevation reflects enhanced oxidative degradation of polyunsaturated fatty acids in cell membranes, likely driven by excessive ROS production (34). Similar findings have been documented in studies showing that exposure to airborne pollutants, including combustion products and particulate matter, leads to elevated MDA levels in rodent lung tissue (35, 36).

Conversely, the activities of CAT and T-SOD—key enzymatic antioxidants involved in ROS detoxification—were significantly suppressed in both candle-exposed groups, with the scented candles exerting a more substantial inhibitory effect. The observed suppression of catalase activity in lung tissue following candle emission exposure is consistent with findings that catalase plays a critical role in defending against oxidative stress induced by environmental pollutants. The more severe decline in the scented candle group may be attributed to the presence of additional synthetic fragrance components, which have been associated with higher VOCs emissions and increased toxicity (6, 37). This proposition is supported by previous research (38) indicating that indoor combustion sources, such as incense burning, are associated with impaired lung function in adolescents. Furthermore, the stability of antioxidant markers in the control group, which was exposed only to fresh air, reinforces the conclusion that the observed oxidative stress is directly attributable to candle-derived emissions.

### 4.4 Molecular and immunohistochemical evidence of pulmonary inflammation

The present study demonstrated that exposure to emissions from both unscented and scented candles significantly upregulated the expression of TNF-*α* and COX-2 in rat lung tissue at both the gene and protein levels. Quantitative analysis showed a duration-dependent increase in mRNA expression, with levels rising progressively from 1 to 6 h per day. These molecular findings were further supported by immunohistochemical staining, which revealed increased localization and intensity of TNF-α and COX-2 proteins in pulmonary tissue, particularly in the scented candle exposed group. Notably, the scented candle group consistently exhibited higher expression levels than the unscented group, suggesting a stronger pro-inflammatory effect likely due to additional fragrance-derived volatile organic compounds (VOCs). TNF-α and COX-2 are key mediators in inflammatory and oxidative stress pathways, and their upregulation reflects activation of pulmonary immune responses (39). These findings are consistent with previous research showing that prolonged exposure to environmental pollutants can elevate glucocorticoid levels, which in turn enhance inflammatory and immune processes. Glucocorticoids regulate gene expression through interaction with transcription factors such as NF-κB, thereby promoting the expression of proinflammatory cytokines (40).

### 4.5 Histological evidence of enhanced lung toxicity from candle exposure

The histopathological results provide strong evidence that candle emissions impair lung structure in a time- and composition-dependent manner. While unscented candles produced progressive injury with longer exposure, scented candles elicited more severe effects even at shorter durations, underscoring their greater toxic potential. The lesions observed—including epithelial degeneration, interstitial fibrosis, and inflammatory infiltration—are consistent with pathological mechanisms reported in studies of indoor air pollution and VOC exposure. Such alterations are closely linked to oxidative stress, persistent inflammation, and airway remodeling, processes that contribute to asthma, chronic obstructive pulmonary disease (COPD), recurrent respiratory infections, and impaired lung development (41). Our findings therefore support growing concerns about the health hazards of indoor combustion sources and emphasize the need for greater awareness and regulation of scented consumer products.

## 5 Conclusion

This study provides compelling evidence that both unscented and scented candles emit a complex mixture of volatile organic compounds (VOCs), as demonstrated by GC–MS analysis. Several of these compounds exhibited unfavorable toxicological profiles in silico, and this was corroborated by biochemical alterations, gene expression changes, increased inflammatory markers, and progressive histopathological damage in exposed rats. Notably, scented candles produced more severe pulmonary injury, including necrosis, fibrosis, and marked inflammatory infiltration, compared with unscented candles and controls. Although these results were obtained under controlled experimental conditions, they highlight the potential toxicological risks of candle emissions and emphasize the role of everyday consumer products in contributing to indoor air pollution. According to the World Health Organization (WHO), 99% of the global population is already exposed to air pollution levels exceeding international guidelines (42). Our findings therefore underscore the urgent need for increased public health awareness and stronger preventive measures. In particular, policy-oriented actions such as regulating emission standards for fragranced products, requiring clear labeling of chemical constituents, and promoting ventilation guidance for indoor spaces would help minimize exposure risks. Together, these steps could reduce the health burden associated with indoor air pollutants and provide evidence-based direction for both consumers and policymakers.

## Data Availability

The datasets presented in this study can be found in online repositories. The names of the repository/repositories and accession number(s) can be found in the article/Supplementary material.

## Glossary

ADMET: Absorption, Distribution, Metabolism, Excretion, and Toxicity
VOC: Volatile organic compound
GC–MS: Gas chromatography–mass spectrometry
SPME: Solid phase microextraction
TNF-*α*: Tumor necrosis factor-alpha
IL-6: Interleukin-6
MDA: Malondialdehyde
CAT: Catalase
T-SOD: Total superoxide dismutase
qRT-PCR: Quantitative real-time polymerase chain reaction
cDNA: Complementary DNA
DAB: Diaminobenzidine
IHC: Immunohistochemistry
H&E: Hematoxylin and eosin
ANOVA: Analysis of variance
CU-IACUC: Cairo University - Institutional Animal Care and Use Committee
ABC: Avidin-biotin complex
ELISA: Enzyme-linked immunosorbent assay
CNS: Central nervous system
ROS: Reactive oxygen species
NF-κB: Nuclear factor kappa-light-chain-enhancer of activated B cells
COPD: Chronic obstructive pulmonary disease
WHO: World Health Organization
LogP: Logarithm of partition coefficient
LogS: Logarithm of solubility
BBB: Blood–brain barrier
CL: Clearance
T½: Half-life
DILI: Drug-induced liver injury
BCF: Bioconcentration factor
QED: Quantitative estimate of drug-likeness
Tox21: Toxicology in the 21st Century
Alarm_NMR: nuclear magnetic resonance-based structural alert
BMS: Bristol-Myers Squibb
CYP: Cytochrome P450 enzymes
