# Supplementary material for: Toxicological evaluation of volatile organic compounds emitted from scented candles: in silico ADMET profiling, oxidative stress, inflammation, and lung injury in rats
Source: Front Public Health. 2025 Sep 18;13:1678549. doi: 10.3389/fpubh.2025.1678549 (PMC12488666; doi:10.3389/fpubh.2025.1678549)
Supplement: Supplementary file 1 [file Data_Sheet_1.pdf]

Table S1: Physicochemical Properties of Unscented Candle Compounds

Name	LogS	LogD	LogP	MW	Vol	Dense	nHA	nHD	TPSA	nRot	nRing	MaxRing	nHet	fChar	nRig	Flex	nStereo
Butane, 2-isothiocyanato-	-1.932	1.649	2.997	115.05	119.269	0.965	1	0	12.36	2	0	0	2	0	2	1	1
1,4,2,5 Cyclohexanetetrol	-0.071	-1.6	-1.879	148.07	138.937	1.066	4	4	80.92	0	1	6	4	0	6	0	4
**Toluene**	-2.364	2.682	2.544	92.06	113.163	0.814	0	0	0	0	1	6	0	0	6	0	0
Heptanal	-1.789	2.055	2.169	114.1	135.782	0.84	1	0	17.07	5	0	0	1	0	1	5	0
Hexanoic acid	-1.245	1.216	1.875	116.08	127.276	0.912	2	1	37.3	4	0	0	2	0	1	4	0
Decane	-5.72	4.411	5.668	142.17	181.516	0.783	0	0	0	7	0	0	0	0	0	inf	0
trans-p-mentha-1(7),8-dien-2-ol	-1.893	2.331	2.232	152.12	176.477	0.862	1	1	20.23	1	1	6	1	0	8	0.125	2
7-Hexadecenal, (Z)-	-3.328	4.025	4.894	238.23	288.81	0.825	1	0	17.07	13	0	0	1	0	2	6.5	0
2(3H)-Furanone, 5-heptyldihydro-	-3.575	2.987	3.644	184.15	205.2	0.897	2	0	26.3	6	1	5	2	0	6	1	1
Undecane	-6.168	4.463	6.169	156.19	198.812	0.786	0	0	0	8	0	0	0	0	0	inf	0
Dodecane	-6.515	4.493	6.66	170.2	216.108	0.788	0	0	0	9	0	0	0	0	0	inf	0
2-Decanone	-3.04	3.32	3.374	156.15	187.67	0.832	1	0	17.07	7	0	0	1	0	1	7	0
Tridecane	-6.763	4.516	7.116	184.22	233.404	0.789	0	0	0	10	0	0	0	0	0	inf	0
2-Undecanone	-3.612	3.616	3.815	170.17	204.966	0.83	1	0	17.07	8	0	0	1	0	1	8	0
Tetradecane	-6.923	4.548	7.565	198.23	250.7	0.791	0	0	0	11	0	0	0	0	0	inf	0
Naphthalene, 1,7-dimethyl-	-4.957	3.848	4.287	156.09	185.813	0.84	0	0	0	0	2	10	0	0	11	0	0
Pentadecane	-7.003	4.594	7.998	212.25	267.996	0.792	0	0	0	12	0	0	0	0	0	inf	0
3-(2-Methyl-propenyl)-1H-indene	-4.733	3.629	4.272	170.11	203.109	0.838	0	0	0	1	2	9	0	0	11	0.091	0
Hexadecane	-7.084	4.64	8.415	226.27	285.292	0.793	0	0	0	13	0	0	0	0	0	inf	0
Heptadecane	-7.165	4.685	8.824	240.28	302.588	0.794	0	0	0	14	0	0	0	0	0	inf	0

Table S 2 : Physicochemical Properties of Scented Candle Compounds

Name	MW	Vol	Dense	nHA	nHD	TPSA	nRot	nRing	MaxRing	nHet	fChar	nRig	Flex	nStereo	LogS	LogD	LogP
Methylene chloride	83.95	56.275	1.492	0	0	0	0	0	0	2	0	0		0	-0.967	0.781	1.262
1,3,5-Cycloheptatriene	92.06	113.163	0.814	0	0	0	0	1	7	0	0	7	0	0	-2.427	1.526	2.473
Bicyclo[2.1.1]hexan-2-ol, 2-ethenyl-	124.09	135.965	0.913	1	1	20.23	1	2	0	1	0	8	0.125	1	-1.683	1.097	1.393
(3-Amino-4,6-dimethylthieno[2,3-b]pyridin-2-yl)(phenyl)methanone	268.07	270.528	0.991	3	2	55.98	2	3	9	4	0	17	0.118	0	-5.122	3.157	3.356
Bicyclo[3.1.1]heptane, 6,6-dimethyl-2-methylene-, (1S)-	136.13	161.767	0.842	0	0	0	0	2	0	0	0	9	0	2	-4.389	3.668	3.625
Cyclohexene, 1-(2-nitro-2-propenyl)-	167.09	176.332	0.948	3	0	43.14	3	1	6	3	0	8	0.375	0	-2.437	2.607	2.571
D-Limonene	136.13	167.687	0.812	0	0	0	1	1	6	0	0	7	0.143	1	-4.107	3.509	4.446
Eucalyptol	154.14	173.194	0.89	1	0	9.23	0	2	0	1	0	9	0	0	-1.918	2.787	2.582
7-Octen-2-ol, 2,6-dimethyl-	156.15	187.67	0.832	1	1	20.23	5	0	0	1	0	1	5	1	-2.326	2.748	2.87
Hexane, 1-chloro-5-methyl-	134.09	144.839	0.926	0	0	0	4	0	0	1	0	0	inf	0	-3.564	3.412	3.816
Isopulegol	154.14	179.114	0.861	1	1	20.23	1	1	6	1	0	7	0.143	3	-2.378	2.963	2.921
Linalool	154.14	185.034	0.833	1	1	20.23	4	0	0	1	0	2	2	1	-2.372	2.426	2.978
2H-Pyran, tetrahydro-4-methyl-2-(2-methyl-1-propenyl)-	154.14	179.114	0.861	1	0	9.23	1	1	6	1	0	7	0.143	2	-2.927	3.106	3.377
Phenylethyl Alcohol	122.07	139.249	0.877	1	1	20.23	2	1	6	1	0	6	0.333	0	-1.196	1.406	1.372
1,2-Dihydrolinalool	156.15	187.67	0.832	1	1	20.23	4	0	0	1	0	1	4	1	-2.398	2.424	3.252
Cyclohexanol, 2-methyl-5-(1-methylethenyl)-, (1.alpha.,2.beta.,5.alpha.)-	154.14	179.114	0.861	1	1	20.23	1	1	6	1	0	7	0.143	3	-2.062	3.02	2.843
Acetic acid, phenylmethyl ester	150.07	162.698	0.922	2	0	26.3	3	1	6	2	0	7	0.429	0	-1.809	1.965	2.034
endo-Borneol	154.14	173.194	0.89	1	1	20.23	0	2	6	1	0	8	0	3	-2.746	3.188	2.968
cis-Ethyl-linalyl acetate	210.16	243.075	0.865	2	0	26.3	7	0	0	2	0	3	2.333	1	-2.891	2.911	2.877
Dodecane	170.2	216.108	0.788	0	0	0	9	0	0	0	0	0	inf	0	-6.515	4.493	6.66
p-Methylbenzyl acetate	164.08	179.994	0.912	2	0	26.3	3	1	6	2	0	7	0.429	0	-2.283	2.591	2.467
Citronellal	154.14	185.034	0.833	1	0	17.07	5	0	0	1	0	2	2.5	1	-2.819	2.945	3.195
Carbonic acid, but-3-yn-1-yl octyl ester	226.16	251.866	0.898	3	0	35.53	11	0	0	3	0	2	5.5	0	-4.162	3.426	4.144
2-Octen-1-ol, 3,7-dimethyl-	156.15	187.67	0.832	1	1	20.23	5	0	0	1	0	1	5	0	-3.247	3.521	3.155
Linalyl acetate	196.15	225.779	0.869	2	0	26.3	6	0	0	2	0	3	2	1	-2.927	2.792	3.295
2,4-Heptadienal, 2,4-dimethyl-	138.1	165.101	0.836	1	0	17.07	3	0	0	1	0	3	1	1	-1.208	1.075	2.022

Cyclohexanemethanol, 4-(1-methylethyl)-, trans-	156.15	181.75	0.859	1	1	20.23	2	1	6	1	0	6	0.333	0	-3.137	3.184	3.208
Cyclohexanemethanol, 4-(1-methylethyl)-, cis-	156.15	181.75	0.859	1	1	20.23	2	1	6	1	0	6	0.333	0	-3.137	3.184	3.208
Bicyclo[2.2.1]heptan-2-ol, 1,7,7-trimethyl-, acetate, (1S-endo)-	196.15	213.939	0.917	2	0	26.3	2	2	6	2	0	9	0.222	3	-2.902	3.165	3.15
Octanal, 7-hydroxy-3,7-dimethyl-	172.15	196.46	0.876	2	1	37.3	6	0	0	2	0	1	6	1	-1.242	1.321	1.292
Bicyclo[4.1.0]heptane, 3,7,7-trimethyl-, [1S-(1.alpha.,3.beta.,6.alpha.)]-	154.14	173.194	0.89	1	1	20.23	0	2	7	1	0	8	0	3	-2.58	2.068	2.533
Limonen-6-ol, pivalate	236.18	269.111	0.878	2	0	26.3	4	1	6	2	0	8	0.5	2	-4.473	4.036	4.511
Diphenyl ether	170.07	191.967	0.886	1	0	9.23	2	2	6	1	0	12	0.167	0	-4.108	3.46	3.598
Caryophyllene	204.19	245.61	0.831	0	0	0	0	2	11	0	0	13	0	2	-5.81	4.756	5.341
exo-7-(2-Propenyl)bicyclo[4.2.0]oct-1(2)-ene	148.13	176.426	0.84	0	0	0	2	2	8	0	0	10	0.2	2	-4.773	3.622	4.276
.beta.-Guaiene	204.19	245.61	0.831	0	0	0	0	2	10	0	0	12	0	2	-4.438	4.359	5.275
3-(4-Isopropylphenyl)-2-methylpropionaldehyde	190.14	223.092	0.852	1	0	17.07	4	1	6	1	0	7	0.571	1	-3.296	3.368	3.383
3-Buten-2-one, 4-(2,6,6-trimethyl-1-cyclohexen-1-yl)-	192.15	225.729	0.851	1	0	17.07	2	1	6	1	0	8	0.25	0	-3.816	2.938	3.535
Retinal	284.21	338.891	0.839	1	0	17.07	5	1	6	1	0	11	0.455	0	-4.987	3.898	4.101
Benzenepropanal, 4-(1,1-dimethylethyl)-	190.14	223.092	0.852	1	0	17.07	4	1	6	1	0	7	0.571	0	-4.093	3.524	3.392
Butylated Hydroxytoluene	220.18	260.321	0.846	1	1	20.23	2	1	6	1	0	6	0.333	0	-5.072	4.154	4.923
3-tert-Butyl-4-hydroxyanisole	180.12	199.927	0.901	2	1	29.46	2	1	6	2	0	6	0.333	0	-2.945	3.267	3.277
2-Methoxy-4-methyl-1-pentylbenzene	192.15	225.729	0.851	1	0	9.23	5	1	6	1	0	6	0.833	0	-5.229	4.255	4.826
Cyclopentaneacetic acid, 3-oxo-2-pentyl-, methyl ester	226.16	245.946	0.92	3	0	43.37	7	1	5	3	0	7	1	2	-2.963	2.121	2.668
Octadecane	254.3	319.884	0.795	0	0	0	15	0	0	0	0	0	inf	0	-7.245	4.731	9.234
Naphthalene, 6,7-diethyl-1,2,3,4-tetrahydro-1,1,4,4-tetramethyl-	244.22	294.862	0.828	0	0	0	2	2	10	0	0	11	0.182	0	-6.779	5.484	6.893
Isopropyl myristate	270.26	317.532	0.851	2	0	26.3	14	0	0	2	0	1	14	0	-6.508	4.637	6.768
Cyclopentadecanone, 2-hydroxy-	240.21	274.384	0.875	2	1	37.3	0	1	15	2	0	16	0	1	-3.214	3.359	4.304
Cyclopenta[g]-2-benzopyran, 1,3,4,6,7,8-hexahydro-4,6,6,7,8,8-hexamethyl-	258.2	295.096	0.875	1	0	9.23	0	3	13	1	0	15	0	2	-6.006	4.271	5.185
Nonadecane	268.31	337.18	0.796	0	0	0	16	0	0	0	0	0	inf	0	-7.327	4.777	9.64
Eicosane	282.33	354.476	0.796	0	0	0	17	0	0	0	0	0	inf	0	-7.402	4.823	10.033
Heneicosane	296.34	371.772	0.797	0	0	0	18	0	0	0	0	0	inf	0	-7.476	4.868	10.427
Docosane	310.36	389.068	0.798	0	0	0	19	0	0	0	0	0	inf	0	-7.548	4.914	10.82
Tricosane	324.38	406.364	0.798	0	0	0	20	0	0	0	0	0	inf	0	-7.621	4.96	11.214

Tetracosane	338.39	423.66	0.799	0	0	0	21	0	0	0	0	0	inf	0	-7.693	5.005	11.607
Bis(2-ethylhexyl) phthalate	390.28	437.082	0.893	4	0	52.6	16	1	6	4	0	8	2	2	-5.291	5.716	7.337
